# Conditions for the spread of CRISPR-Cas immune systems into bacterial populations

**DOI:** 10.1093/ismejo/wrae108

**Published:** 2024-06-19

**Authors:** Josie F K Elliott, David V McLeod, Tiffany B Taylor, Edze R Westra, Sylvain Gandon, Bridget N J Watson

**Affiliations:** Milner Centre for Evolution, Department of Life Sciences, University of Bath, Claverton Down, Bath BA2 7AY, United Kingdom; ESI, Biosciences, University of Exeter, Cornwall Campus, Penryn TR10 9FE, United Kingdom; Département de mathématiques et statistique, Université de Montréal, Montréal, Canada; Institute of Ecology and Evolution, Universität Bern, Bern, Switzerland; Milner Centre for Evolution, Department of Life Sciences, University of Bath, Claverton Down, Bath BA2 7AY, United Kingdom; ESI, Biosciences, University of Exeter, Cornwall Campus, Penryn TR10 9FE, United Kingdom; CEFE, CNRS, Univ Montpellier, EPHE, IRD, Montpellier, France; ESI, Biosciences, University of Exeter, Cornwall Campus, Penryn TR10 9FE, United Kingdom

**Keywords:** CRISPR-Cas, bacteria–phage interactions, mathematical theory, microbial ecology and evolution, evolutionary epidemiology, horizontal gene transfer, ecology, resistance evolution

## Abstract

Bacteria contain a wide variety of innate and adaptive immune systems which provide protection to the host against invading genetic material, including bacteriophages (phages). It is becoming increasingly clear that bacterial immune systems are frequently lost and gained through horizontal gene transfer. However, how and when new immune systems can become established in a bacterial population have remained largely unstudied. We developed a joint epidemiological and evolutionary model that predicts the conditions necessary for the spread of a CRISPR-Cas (clustered regularly interspaced short palindromic repeats–CRISPR-associated) immune system into a bacterial population lacking this system. We found that whether bacteria carrying CRISPR-Cas will spread (increase in frequency) into a bacterial population depends on the abundance of phages and the difference in the frequency of phage resistance mechanisms between bacteria carrying a CRISPR-Cas immune system and those not (denoted as ${f}_{\Delta }$). Specifically, the abundance of cells carrying CRISPR-Cas will increase if there is a higher proportion of phage resistance (either via CRISPR-Cas immunity or surface modification) in the CRISPR-Cas–possessing population than in the cells lacking CRISPR-Cas. We experimentally validated these predictions in a model using *Pseudomonas aeruginosa* PA14 and phage DMS3vir. Specifically, by varying the initial ratios of different strains of bacteria that carry alternative forms of phage resistance, we confirmed that the spread of cells carrying CRISPR-Cas through a population can be predicted based on phage density and the relative frequency of resistance phenotypes. Understanding which conditions promote the spread of CRISPR-Cas systems helps to predict when and where these defences can become established in bacterial populations after a horizontal gene transfer event, both in ecological and clinical contexts.

## Graphical Abstract

**Graphical Abstract:**
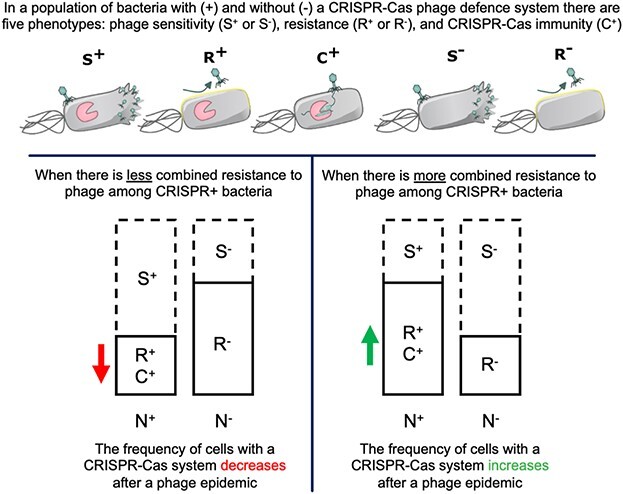

## Introduction

Prokaryotes possess a multitude of defence systems to protect them against invasion by foreign genetic elements such as viruses (bacteriophage/phage), plasmids, or transposons [1]. CRISPR-Cas (clustered regularly interspaced short palindromic repeats–CRISPR-associated) defence systems act in an analogous manner to the mammalian adaptive immune system by “remembering” previous infections and arming the system against reinfections (reviewed in [2]). The process of CRISPR-Cas immunity is carried out by genes in the CCas operon. Cas proteins recognise foreign genetic material and integrate it as ~30–base-pair DNA fragments (termed spacers) into the host genome between short palindromic repeated sequences (termed repeats) at the CRISPR loci [3]. This sequence of events creates a heritable memory of infection. These spacers are transcribed and processed into crRNAs (CRISPR RNAs) and incorporated into a ribonucleoprotein complex with Cas proteins. Re-invading genetic elements are identified by complementary base-pairing with crRNAs, ultimately leading to cleavage of the recognised foreign DNA or RNA by the Cas proteins [2, 4].

Despite the potential benefits a CRISPR-Cas system can offer a host, the prevelance of this defense system varies in its distribution. It is estimated that around 90% of archaea but only 40% of bacteria contain a CRISPR-Cas system [5, 6]. Within bacteria CRISPR-Cas systems are more prevalent in thermophiles [7] but virtually devoid from some major lineages [8]. This distribution may be partly explained by the numerous other defence systems available to bacteria [1]. However, another contributing factor may be that retaining a CRISPR-Cas system incurs costs upon the host. In *Streptococcus thermophilus,* Cas protein expression, as well as activation of the CRISPR-Cas system, had an associated fitness cost [9], whereas in *Pseudomonas aeruginosa* the CRISPR-Cas presence did not have a baseline fitness cost but instead an inducible cost associated with phage infection [10] due to the expression of phage genes prior to immune clearance [11]. CRISPR-Cas systems additionally carry the risk of acquiring host-targeting spacers, resulting in auto-immunity, or spacers that become genome targeting upon integration of foreign elements such as temperate phage [2, 12, 13]. Retaining a CRISPR-Cas system may also incur costs by preventing the co-occurrence or acquisition of potentially beneficial traits. For example, negative co-occurrence is seen between CRISPR-Cas systems and some double-strand break repair systems [14, 15], and CRISPR-Cas systems can compromise the capability of a host cell to acquire beneficial plasmids by targeting these mobile genetic elements [2, 16].

Furthering our understanding of the patchy distribution of CRISPR-Cas systems, laboratory studies have observed that strong selective pressures against CRISPR-Cas systems can lead to bacteria losing or degrading these genes [12, 13, 17–21]. This discovery has led to a proposal that the presence of CRISPR-Cas systems in prokaryote populations may be in a continuous state of flux—termed the “pan immune model” [22]. In this model, when the fitness costs become too great CRISPR-Cas systems are lost or degraded. However, CRISPR-Cas systems are also frequently re-acquired by horizontal gene transfer, re-establishing the defence system in the population. This model has been supported by phylogenetic evidence [6, 23, 24] and some experimental evidence [25].

To gain insights into whether the pan immune model can help to explain CRISPR-Cas distribution, we must first understand the selective pressures that would allow a CRISPR-Cas system to become established and spread when such systems are rare. However, experimental evidence demonstrating the ability of bacteria carrying a CRISPR-Cas system to proliferate and spread in a naïve population is currently lacking [26]. Studies using the opportunistic human pathogen *P. aeruginosa* (PA14) and its phage DMS3vir (a temperate phage [27] modified to be obligately lytic [28]) have shown that there are relatively narrow conditions whereby the type I-F CRISPR-Cas system is favoured as a defence strategy. Growth in nutrient-rich media, or other methods that increased phage exposure and infection risk, led to the populations of *P. aeruginosa* being more likely to evolve resistance via mutation of the type IV pilus that acts as the receptor for DMS3vir [10]—generally described as “surface modification”. A number of other experimentally tested conditions have also shown a preference in *P. aeruginosa* for alternative mechanisms of phage resistance (namely surface modification) over CRISPR-Cas. These conditions include use of strains with high mutation rates [29]; high levels of immigration of naïve, uninfected hosts; [30]; or higher phage diversity [31]. Conversely, more limited examples have shown selection for CRISPR-Cas system usage by *P. aeruginosa,* such as in the context of a mixed microbial species community [32].

Experimental work on how bacteria with CRISPR-Cas systems spread in populations has also been supported by computational models, which predict that when bacteria can evolve surface-modification resistance to lytic phage, the conditions are restricted under which CRISPR-Cas resistance is able to provide bacteria with a selective advantage and become the dominant mechanism for immunity [26, 33, 34]. Bacteria with CRISPR-Cas immunity were predicted to have the ability to dominate the population only in the presence of very low resource concentrations, when surface modification carried a significant fitness cost, mutation rate to resistance was low, and most prominently, when the number of spacers required to end the evolutionary arms race with phage protospacer mutations was reduced [34].

Given that experimentally *P. aeruginosa* will more frequently evolve surface-modification based phage resistance over CRISPR-Cas immunity, as well as the limited conditions for successful spread of CRISPR-Cas hosts in populations according to computational models, further work is needed to better understand when CRISPR-Cas systems are beneficial following frequent horizontal gene transfer (HGT) events. In this study we explored the conditions needed for CRISPR-Cas cells to spread in a population lacking this defence system and considered how these conditions depend on the presence of alternative phage resistance phenotypes in the bacterial populations. We developed a deterministic model to capture this selection process. We found that the difference in the frequency of cells with phage defence (either surface-modification resistance or CRISPR-Cas immunity) in the CRISPR-Cas–possessing subpopulation and the frequency of cells with phage defence (surface-modification resistance) in the subpopulation lacking CRISPR-Cas determines whether CRISPR-Cas subpopulations are selected for or against in the presence of phage. We then validated this mathematical prediction experimentally using the *P. aeruginosa* PA14 and DMS3vir phage model system.

## Materials and methods

### Modelling

To understand the conditions under which CRISPR-Cas immune systems increase or decrease in frequency within a population, we first built a deterministic mathematical model tracking the population dynamics of the 5 bacterial genotypes that can be associated with the mixing of isogenic populations of bacteria with and without CRISPR-Cas systems (described in Table 1).

**Table 1:** Description of each phenotype present in a mixed population with and without CRISPR-Cas systems.

*S* ^+^ *(t)*	Density of bacteria at time *t* that possess a CRISPR-Cas system but are sensitive to phage infection due to a lack of phage targeting spacers
*R* ^+^ *(t)*	Density of bacteria at time *t* that possess a CRISPR-Cas system but lacks phage targeting spacers and have gained resistance to phage infection via mutation of phage targeted receptors on the cell surface (termed “resistance”)
*C* ^+^ *(t)*	Density of bacteria at time *t* that possess a CRISPR-Cas system with a phage targeting spacer and are thus immune to phage infection (termed “immunity”)
*S^−^ (t)*	Density of bacteria at time *t* lacking a CRISPR-Cas system that are sensitive to phage infection
*R^−^ (t)*	Density of bacteria at time *t* lacking a CRISPR-Cas system that are resistant to phage infection via mutation of phage targeted receptors on the cell surface (termed “resistance”)

We assumed that in the absence of phage, bacterial cell division occurs via logistic growth, with a maximum per capita growth rate of *r* and a population carrying capacity of *K*, with bacteria cells dying at a per capita rate of ${\mathcal{m}}_{\,b}$. In the presence of free-living phage, whose density at time *t* is *V(t)*, infection of bacterial cells occurs via mass action with rate constant *α*. Following infection, sensitive bacteria cells (*S*) “burst”, producing *B* free-living phage. Resistance to phage can be acquired through mutation (*R*), which occurs with probability *μ* per cell division, and cells possessing a CRISPR-Cas system can also acquire a phage-targeting spacer during infection (*C*) with probability *A*. The costs of surface-based resistance and CRISPR-Cas immunity are *c_R_* and *c_I_*, respectively, and these costs reduce the cell division rate. Finally, free-living phage die at a per capita rate ${\mathcal{m}}_{\,b}$. Under these assumptions the population dynamics are described by the set of equations:


$$ \frac{d{S}^{-}}{dt}=\left(r\left(1-\frac{N}{K}\right)\left(1-\mu \right)-\alpha V-{\mathcal{m}}_{\,b}\right){S}^{-} $$

$$ \frac{d{R}^{-}}{dt}=\mu r\left(1-\frac{N}{K}\right){S}^{-}+\left(r\left(1-\frac{N}{K}\right)\left(1-{c}_R\right)-{\mathcal{m}}_{\,b}\right){R}^{-} $$

$$ \frac{d{S}^{+}}{dt}=\left(r\left(1-\frac{N}{K}\right)\left(1-\mu \right)-\alpha V-{\mathcal{m}}_{\,b}\right){S}^{+} $$

$$ \frac{d{R}^{+}}{dt}=\mu r\left(1-\frac{N}{K}\right){S}^{+}+\left(r\left(1-\frac{N}{K}\right)\left(1-{c}_R\right)-{\mathcal{m}}_{\,b}\right){R}^{+} $$

$$ \frac{d{C}^{+}}{dt}=\alpha AV\ {S}^{+}+\left(r\left(1-\frac{N}{K}\right)\left(1-{c}_I\right)-{\mathcal{m}}_{\,b}\right){C}^{+} $$

(1) \begin{equation*} \frac{dV}{dt}=\left(\alpha B\ \left({S}^{-}+{S}^{+}\left(1-A\right)\right)-{\mathcal{m}}_{\,v}-\alpha \left({S}^{+}+{S}^{-}+{C}^{+}\right)\right)V \end{equation*}

where $N$ = ${S}^{-}$ + ${R}^{-}$ + ${S}^{+}$ + ${R}^{+}$ + ${C}^{+}$ is the total bacteria population density at time *t*.

### Bacterial and phage strains

All strains used were derived from *P. aeruginosa* UCBPP-PA14. The phage DMS3vir was used in all experiments (where phage were added). The strain used to represent sensitive CRISPR+ cells (*S^+^*) was the UCBPP-PA14 wild-type strain, which contains a CRISPR-Cas system but does not have fully matching spacers to provide immunity against DMS3vir. These spacers are described as “primed” spacers—spacers that partially match the phage genome but are not capable of efficiently targeting the phage to provide immunity. A spontaneous surface-receptor mutant derived from the wild-type strain was used as the CRISPR+ phage–resistant mutant strain (*R*^+^) (SM-1). The CRISPR+ immune strain (*C^+^*) contained two spacers that targeted DMS3vir to provide immunity against the phage BIM2 [35]. A functional CRISPR-Cas knockout was used to represent a phage- sensitive population lacking a CRISPR-Cas system—PA14 *csy3::lacZ* (*S^−^*) [28] (the *lacZ* gene disrupts an essential gene in the CRISPR-Cas system and renders it nonfunctional). From this engineered strain, a spontaneous surface-receptor mutant was used as the CRISPR–phage resistant mutant (*csy3::lacZ*-SM) (*R^−^*) [10]. This surface mutant was generated independently of the SM-1 spontaneous mutant (*R^+^*). For schematic representation of strains used see Fig. 2a.

### Tn7 modification of strains

To allow easy strain identification by quantitative polymerase chain reaction (qPCR) amplification, each strain was genetically tagged with short noncoding gene blocks (107–158 bp) ordered from IDT (Table 2). These gene blocks were incorporated into the pUC18T-mini-Tn7T-Gm plasmid (pUC18T-mini-Tn7T-Gm was a gift from Herbert Schweizer – Addgene plasmid number 63121; http://n2t.net/addgene:63121; RRID:Addgene_63 121) via restriction-ligation cloning using HindIII and SpeI (New England Biosciences). The gene block DNA was inserted into the genome of each *P. aeruginosa* strain using four-parent conjugal puddle mating in a version of the protocol from [36], using the helper strains SM10(*λpir*)/pTNS2 and HB101/pRK2013. The presence of the gene block DNA at the att-Tn7 site in the *P. aeruginosa* genome was confirmed by PCR amplification and Ssanger sequencing.

**Table 2:** Sequences and primers used for genetically tagging strains for qPCR quantification.

Strain of PA14	Gene block added	Primers used in qPCR amplification
*S^+^* − WT	ATCAGAATGCCGCGGTGAATACGTTCCCGGGCCTTGTACACACCGCCCGTCACACCATGGGAGTTTGTTGCACCAGAAGTAGCTAGCCTAACTGCAAAGAGGGCGGT	WT-F: ATCAGAATGCCGCGGTGAAT
		WT-R: ACCGCCCTCTTTGCAGTTAG
*R^+^* − SM-1	GGGATGACGGTACCGGAAGAATAAGCACCGGCTAACTACGTGCCAGCAGCCGCGGTAATACGTAGGGTGCGGGCGTAAAGCGTGCGCAGGCGGTTTGCTAAGACCGATGTGAAATCCCCGGGCTCAACCTGGGAACTGCATTGGTGACTGGCAGGCTA	SM1-F: GGGATGACGGTACCGGAAGA
		SM1-R: TAGCCTGCCAGTCACCAATG
*C^+^* − BIM2	TCGCCACCCTTATTGCCATTATTTTTCTCACACTATTTGCGCGGCGCCAACGTCGTCAAACGCATCCAATTGTCGACTCTTTCTATGGTGATCATCGTGGGTATTGAATTTCTGCTAAGCCAACGCTTACAGTTAGTCGCCG	BIM2-F: TCGCCACCCTTATTGCCATT
		BIM2-R: CGGCGACTAACTGTAAGCGT
*S^−^* − *csy3::lacZ*	GTTTATGGAACACCATGTGATAATGAGGCAATACAAAGAAATTGCAGATAAATATCATCTATATAAGAATCACGATTATAAAGAAATATGATCTTAGTATATCTAAAAGCATATCAGATAATGTTATTTGTTTGCC	*csy3lacZ*-F: GTTTATGGAACACCATGTGATAATG
		*csy3lacZ*-F: GGCAAACAAATAACATTATCTGATATGC
*R^−^* − c*sy3::laZ-*SM	TCATTTTTGACATGAAGAGAAACATCGATAAAAGGATGCTCGTTAAAGAAAGATTTTAAAAATTTGGGCATAATGAATGTCGCGATATATGAAGACACGACAACATTTAATTTCGA	*csy3lacZ*SM-F: TCATTTTTGACATGAAGAGAAACAT
		*csy3lacZ*SM-R: TCGAAATTAAATGTTGTCGTGTCTTC

### Competition experiments

Experiments were performed in 6 independent replicates. Strain competition was performed in 6-ml glass vials of minimal M9 media—22 mM Na_2_HPO_4_, 22 mMKH_2_PO_4_, 8.6 mM NaCl, 20 mM NH_4_Cl, 1 mM MgSO_4_, and 0.1 mM CaCl_2_ supplemented with 0.2% glucose. This study focused on competition between “pre-evolved” strains that already possess either immunity or resistance; previous studies have shown that low resource conditions select for CRISPR-Cas–based immunity over surface modification–based resistance in this model system [10]. The respective strains were grown separately for 48 hours (including a 1:100 transfer after 24 hours) in vials of 6 ml M9 media before being mixed in the ratios indicated. These mixed cultures were used to inoculate vials at 1:100 with no additional phage, or with 10^4^ pfu/ml phage added. Cultures were incubated at 37°C with shaking at 180 rpm for 24 hours.

Total cell counts were determined by spot plating serially diluted culture (2 μl). Phages were extracted using chloroform extraction (sample: chloroform 10:1 v/v) and phage titres were then determined by spotting serial dilution of isolated phage samples in phosphate-buffered saline on a lawn of PA14 *csy3::lacZ*. Samples to which phage were not added were also sampled for phage and those with phage contamination were removed from downstream analysis.

### DNA extraction and qPCR amplification

The densities of the different resistant types of *P. aeruginosa* PA14 at the start and end of the experiment were determined using qPCR amplification, which has been previously validated [32]. Genomic DNA was extracted from initial culture mixes and final 24-hour grown cultures using the Dneasy UltraClean Microbial Kit (Qiagen), following the manufacturer’s instructions. Primers used are listed in Table 1. The amplification reactions were performed with Brilliant SYBR Green reagents (Agilent) in 20-μl reactions with 10 μl master mix, 2 μl primer pair, 0.4 μl dye, and sterile nuclease-free water to a total volume of 15 μl before adding 5 μl of DNA (each genome extraction was diluted 1:5 in nuclease-free water). Amplification reactions were made in triplicate. The qPCR amplification program was performed at 95°C for 3 min at 40 cycles at 95°C for 10 s and 60°C for 30°s. The Applied Biosystems QuantStudio 7 Flex Real-Time PCR system machine and associated software were used to analyse all qPCR amplification reaction results.

All statistical analyses were performed in the R statistical package v 4.0.3 [37]. The association between the change in CRISPR+ cell population and the initial conditions of the experiment were assessed using a general linear model, with bootstrapped confidence intervals reported.

## Results

### Mathematical model of the conditions for the spread of CRISPR-Cas

Our objective was to understand the evolutionary dynamics of CRISPR-Cas immune systems in a bacterial population. When CRISPR-Cas immune systems are the only possible way to defend against phage, the CRISPR-Cas–containing subpopulation would increase in frequency within the defenceless population in the presence of phage, providing the costs are not too high (not shown). If bacteria are already resistant to the phage (for example because they carry an alternative defence system) CRISPR-Cas would never increase in frequency in the population (not shown). However, it is less obvious whether CRISPR-Cas systems increase, or decrease, in frequency if bacteria are sensitive to phage but may also acquire mutations that prevent phage infection; moreover, it is not clear what role the initial frequency of CRISPR-Cas plays. Previous modelling revealed that the lower the initial frequency of the CRISPR-Cas population, the less likely the population is to become established [26]. However, the deterministic population dynamic model defined by equations (1, see Methods) predicts that the initial frequency of the bacteria with CRISPR-Cas systems will not determine whether they increase or decrease in frequency in the short term. To explore what affects the change in CRISPR frequency, we defined ${N}^{+}(t)\equiv{S}^{+}(t)+{R}^{+}(t)+{C}^{+}(t)/N(t)$ as the frequency of CRISPR^+^ bacteria cells and



^(2)^
\begin{equation*} {f}_{\Delta }\equiv \frac{\ {R}^{+}+{C}^{+}}{S^{+}+{R}^{+}+{C}^{+}}-\frac{\ {R}^{-}}{R^{-}+{S}^{-}} \end{equation*}

as the difference in frequency of resistance or immunity in CRISPR^+^ cells vs CRISPR*^−^* cells (see Table 1 for *S*^+^, *R*^+^, *C*^+^, *R*^−^, *S*^−^ descriptions). Supposing that costs of immunity or resistance are negligible (*c_R_ ≈* 0, *c_I_ ≈* 0) and mutations and acquiring resistance or immunity is rare (*μ ≈* 0, *A ≈* 0), then from equation (1) we obtain


(3) \begin{equation*} \frac{d{N}^{+}}{dt}=\alpha V{f}_{\Delta }{N}^{+}\left(1-{N}^{+}\right) \end{equation*}

**Figure 1:**
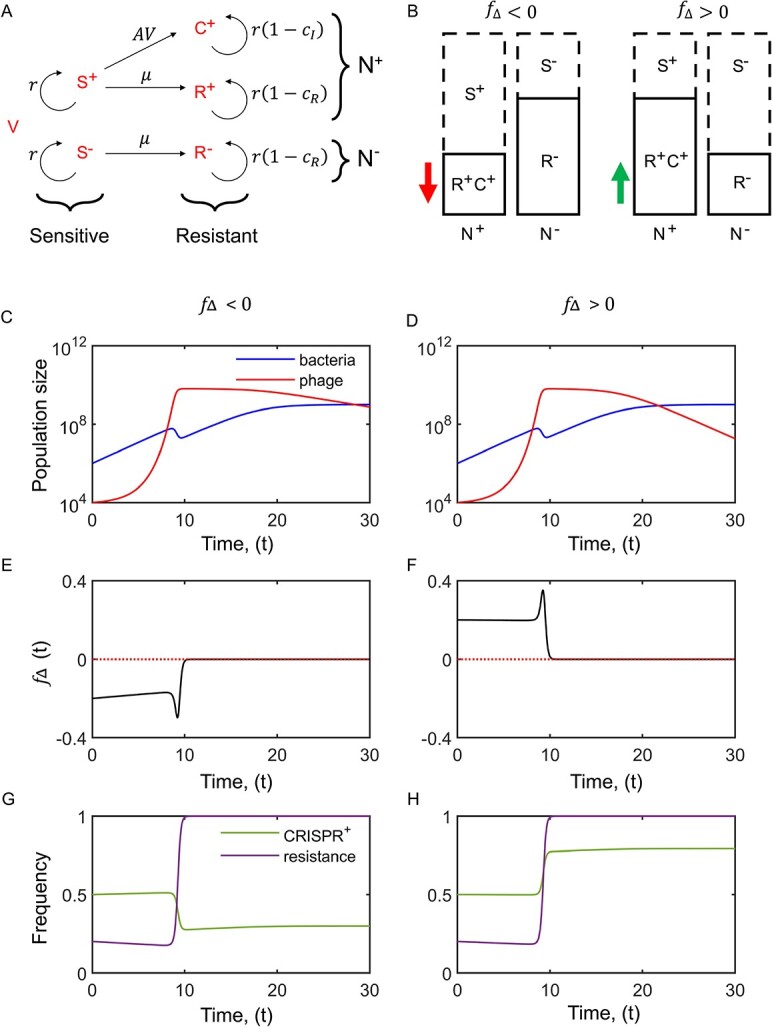
(A) The 5 different types of cells differ in their phenotype (susceptible, S, or resistant to the virus, *V*) and in their genotype (N+ is the frequency of cells in the population carrying CRISPR, divided by the total population density, and N− is the frequency of cells that do not carry CRISPR, divided by the total population density). Resistance (to broadly describe phenotypes that make the cell non-susceptible to phage predation) includes both surface receptor-based resistance (R) and CRISPR-Cas immunity based resistance (C). The evolution of CRISPR-Cas immune cells, C, depends on viral infection, *V*, and the probability of spacer acquisition, *A*, whereas surface modification, R, depends on the mutation rate, $\mu$ (both *A* and $\mu$ are predicted to be negligible due to strong selection imposed by the phage epidemic, see parameter values below). (B) Effect of ${f}_{\Delta }$ on the change in frequency of CRISPR after a phage epidemic [see equation (3)]: the phage epidemic removes the susceptible cells (dashed rectangle) and changes the frequency of CRISPR if ${f}_{\Delta }$ ≠ 0. When ${f}_{\Delta }$ < 0 (red arrow, pointing downwards) the frequency of CRISPR after the phage epidemic drops. In contrast, when ${f}_{\Delta }$ >0 (green arrow, pointing upwards) the frequency of CRISPR after the phage epidemic increases. (C–H) Illustration of example bacteria and phage population dynamics: (C,D), behaviour of ${f}_{\Delta }(t)$ (E,F)*,* and change in frequency of resistance ((${R}^{-}(t)+{R}^{+}(t)+{C}^{+}(t))/N(t)$) and CRISPR+ cells (${N}^{+}(t))$ (G,H) when ${f}_{\Delta }<0$ (c,e,g) and when ${f}_{\Delta }>0$ (D,F,H). Because cells that are CRISPR+ (with two phage spacers) remove phage from the population, when there is a greater frequency of resistance in CRISPR+ cells (${f}_{\Delta }>0$), this will tend to cause a more rapid decline in phage (compare panels c and d), especially at low densities. Parameter values: $r=0.5, {{m}}_{\,b}={{m}}_{\,v}=0,K={10}^9,\alpha ={10}^{-9},\mu =0,B=100,{c}_R=0.05,{c}_I=0,A=0$. All panels assumed initially 50% of bacteria cells are CRISPR+, and that 50% of CRISPR+ cells with resistance have a genetic mutation (R^+^) (so 50% have resistance through CRISPR, C^+^) and that the initial densities of bacteria and virus are $N(0)={10}^6$and $V(0)={10}^4$, respectively.

Thus, in the deterministic model, what determines whether CRISPR+ increases or decreases in frequency is given by the equation for $\frac{d{N}^{+}}{dt}$, whose sign depends on ${f}_{\Delta }$ and not the frequency of CRISPR+ cells. When ${f}_{\Delta }>0$, the frequency of cells possessing a CRISPR-Cas system will increase in the population, and when ${f}_{\Delta }<0$, the frequency of CRISPR+ cells will decrease. As might be expected, this effect also depends on the abundance of phage. If there are no phage, *V(t)* = 0; then in the absence of the costs of CRISPR, $\frac{d{N}^{+}}{dt}=0$, there will be no change in the frequency of the CRISPR+ population. If *V(t)* > 0 (there are phage), then CRISPR+ cells will increase or decrease in frequency depending on whether ${f}_{\Delta }>0$ or ${f}_{\Delta }<0$. In the presence of phage, we would expect that in the absence of costs, resistance will go to fixation. At this point, ${f}_{\Delta }=0$ (provided there is some resistance present on both CRISPR+ and CRISPR−- cells) and so the frequency of CRISPR+ cells will no longer change (Fig. 1). However, before this occurs, ${f}_{\Delta }$will always have the same sign, and so CRISPR+ cells will monotonically increase or decrease depending upon the initial value of ${f}_{\Delta }$ (Fig. 1). Simulation results indicate that the same conclusions hold in the presence of costs of both resistance and immunity, provided these costs are not too large (Supplementary Fig. S1) The rate of increase (or decrease) in the frequency of CRISPR+ cells (*N*^+^) will be proportional to *V(t)*. Thus, when understanding equation (3), the selection coefficient for CRISPR+ cells is $\alpha V{f}_{\Delta }$, and therefore the magnitude of the selection coefficient will depend upon the magnitude of $\alpha$, $V$, and ${f}_{\Delta }$, whereas the sign of the selection coefficient will depend on the sign of ${f}_{\Delta }$ (assuming $V$, $\alpha$ > 0). If we imagine a subpopulation of invading cells that possess a CRISPR-Cas system where, for example, 30% of this population shows phage resistance (either by CRISPR-Cas immunity or surface modification of phage receptors), and the subpopulation lacking CRISPR-Cas only has 15% of the cells with a phage resistance phenotype (that is, through surface modification)—then in the presence of phage, the CRISPR-Cas–possessing subpopulation of cells will be selected for and will increase in frequency in the total population. This remains the case even if the absolute number of cells in the CRISPR− resistance population is larger than the absolute number of resistant cells in the CRISPR+ population. Therefore, the initial frequency of cells with a CRISPR-Cas system does not determine the direction of selection, rather it is the relative difference between the proportions of the populations that have a phage protective phenotype, as described by ${f}_{\Delta }$.

**Figure 2:**
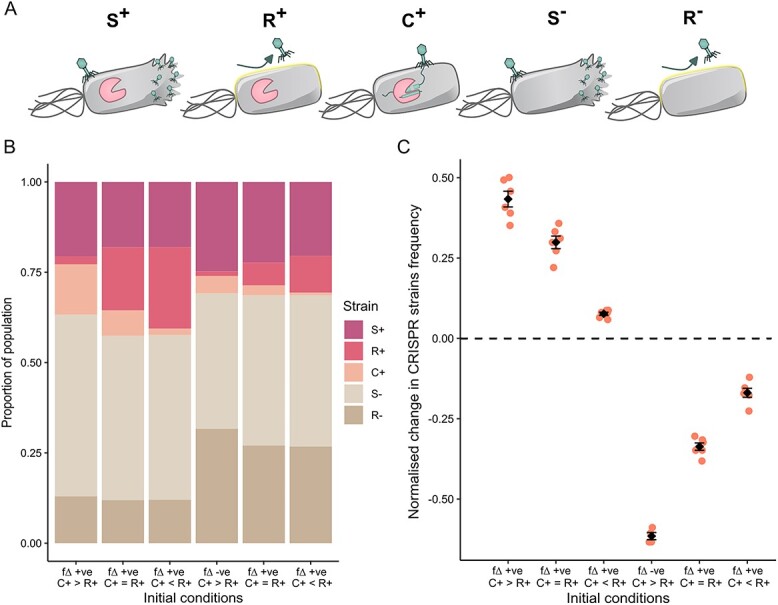
(A) Schematic representing each genotype of *P. Aeruginosa* PA14 present in the mixed population in the model (S: Phage sensitive, R: Resistant via surface modification, C: CRISPR-Cas based immunity, + carry a CRISPR-Cas system, − do not carry CRISPR-Cas). (B) Stacked bar chart showing the average relative frequencies of each strain in the initial population mixes (N = 6). Initial population phenotype mixes are fully described in table 3, with ${f}_{\Delta }>0$ describing when proportion of the CRISPR+ population with protective phenotypes against the phage is greater than the proportion in the CRISPR− population, and vice versa for ${f}_{\Delta }$ < 0. This is explored in conditions where the number of CRISPR immune cells in the CRISPR+ population (C+) is either greater “>”, equal “=”, or less than “<” the number of cells with surface-based resistance (R+). Colours represent the different strain genotypes. (c) the change in frequency of the CRISPR+ population (${N}^{+}(t)=$$\left({S}^{+}(t)+{R}^{+}(t)+{C}^{+}(t)\right)/N(t)$) after 24 hours co-cultured together and in the presence of phage as compared to the same initial mix not under phage selection (frequency with phage – frequency without phage)/frequency without phage). N = 6, circles represent individual replicates with the black diamond showing the mean and the standard error bars representing the interquartile range.

We did not consider HGT in our model because it is likely rare during phage epidemics (relative to the strength of selection). However, mathematical analysis of the implications of HGT for the spread of CRISPR suggests that it can have both a positive and a negative impact on ${f}_{\Delta }$, depending on donor and recipient identities. For example, if resistance (either mutations for surface modification, R, or spacer acquisition for CRISPR-Cas, C) is rare, as might be expected in the early part of a phage epidemic, then HGT will be more likely to induce *S^−^* to S+ transitions, and HGT will have a net negative effect on ${f}_{\Delta }$ (see SI). Once general resistance (either R or C) has become more abundant in the population, *S^−^* to *C*^+^, or *R^−^* to *R*^+^ transitions may also be common, depending upon whether resistance is more commonly linked to CRISPR, or if it is genetic, and in this case HGT can have a net positive or negative impact on ${f}_{\Delta }$ transitions (see SI).

### Empirical testing supports the deterministic model that the sign of ${f}_{\Delta }$prescribes whether CRISPR cells are selected for or against in a mixed population

To understand the relevance of our deterministic model to bacterial populations, we empirically tested the predictions. Namely, that when ${f}_{\Delta }$ is positive (the frequency of resistant or immune phenotype cells in the CRISPR+ subpopulation is higher than the frequency of resistance phenotype cells in the CRISPR−- subpopulation) the frequency of CRISPR+ cells (${N}^{+}$) will increase, and vice versa if ${f}_{\Delta }$ is negative. To empirically test the deterministic model above, we competed 5 strains of *P. aeruginosa* PA14 to represent each of the 5 genotypes in the model. Combining the 5 strains at different initial frequencies allowed us to set up initial conditions where the value of ${f}_{\Delta }$ was either positive or negative. By genetically tagging each strain, their relative proportions could be determined using qPCR amplification after 24 hours of competition in either the presence or absence of phage selection pressure from the lytic phage DMS3vir.

Each of the 5 genotypes described by the above model were represented with a different strain of *P. aeruginosa* PA14 (schematic Fig. 2a): wild type for the *S*^+^ phage-sensitive, CRISPR+ population of cells; a spontaneous phage receptor mutant for the *R*^+^ phage-resistant, CRISPR+ population cells; a strain with 2 DMS3vir phage–targeting spacers in the CRISPR array for the *C*^+^ population of CRISPR+, phage-immune cells. The CRISPR−- phage-sensitive population of *S^−^* cells, was represented by a functional knockout of the CRISPR-Cas system via insertion of a *lacZ* gene at the *cas3* locus, and the *R^−^* phage-resistant population of CRISPR− cells was represented by a spontaneous phage receptor mutant of the CRISPR-Cas knockout strain. Cells with both CRISPR-Cas immunity and surface-based resistance to the phage were not included because the emergence of this genotype experimentally is rare [10]. Indeed, the possession of both defence strategies offers no additional fitness benefit to the cell as each individual resistance confers almost complete protection against phage infection [38].

Using the described strains as examples of the 5 genotypes described by the deterministic model allows the predictions to be tested over a short, 24-hour selection experiment to observe how the CRISPR+ cells (*S*^+^ + *R*^+^ + *C*^+^) change in frequency in the population. Due to the short timescale of the experiment we would expect minimal transitions between different genotypes via either acquisition of additional spacers (*A*) or mutation of surface receptors (*μ)*. Initial mixes of strains were created (Table 3) with three conditions where ${f}_{\Delta }$ is initially positive and three where ${f}_{\Delta }$ is initially negative. Within that, initial mixes were also varied to create conditions where the CRISPR+ immune cells greatly outnumbered (*C*^+^ > *R*^+^), were approximately equal to (*C*^+^ = *R*^+^), or were outnumbered by (*C*^+^ < *R*^+^) the surface-based resistant cells (Fig. 2b). The relative frequencies of each genotype were measured via qPCR amplification at the beginning and end of 24 hours of co-culturing, both with and without phage selection. The overall population reached similar cell densities in both the phage and no-phage treated runs (Supplementary Fig. S2a), and the phage titre increased similarly across all initial strain mixes (Supplementary Fig. S2b) with only a slight disparity in the phage titre increase in the ${f}_{\Delta }$-positive, *C*^+^ < *R*^+^ condition.

**Table 3:** Description of the six initial conditions used in the short-term evolution experiment.

Initial mix of strains	Description of phenotype ratios
${f}_{\Delta }$ positive (+ve), (*C*^+^ > *R*^+^)	The proportion of the CRISPR+ population with protective phenotypes against the phage, is greater than the proportion in the CRISPR− population. Within the CRISPR+ population there are more CRISPR immune cells than surface-based resistant cells.
${f}_{\Delta }$ positive (+ve), (*C*^+^ = *R*^+^)	The proportion of the CRISPR+ population with protective phenotypes against the phage, is greater than the proportion in the CRISPR− population. Within the CRISPR+ population there are approximately equal CRISPR immune cells and surface-based resistant cells.
${f}_{\Delta }$ positive (+ve), (*C*^+^ < *R*^+^)	The proportion of the CRISPR+ population with protective phenotypes against the phage, is greater than the proportion in the CRISPR− population. Within the CRISPR+ population there are fewer CRISPR immune cells than surface-based resistant cells.
${f}_{\Delta }$ negative (−ve), (*C*^+^ > *R*^+^)	The proportion of the CRISPR+ population with protective phenotypes against the phage, is less than the proportion in the CRISPR− population. Within the CRISPR+ population there are more CRISPR immune cells than surface-based resistant cells.
${f}_{\Delta }$ negative (−ve), (*C*^+^ = *R*^+^)	The proportion of the CRISPR+ population with protective phenotypes against the phage, is less than the proportion in the CRISPR− population. Within the CRISPR+ population there are approximately equal CRISPR immune cells and surface-based resistant cells.
${f}_{\Delta }$ negative (−ve), (*C*^+^ < *R*^+^)	The proportion of the CRISPR+ population with protective phenotypes against the phage, is less than the proportion in the CRISPR− population. Within the CRISPR+ population there are fewer CRISPR immune cells than surface-based resistant cells.

Assuming negligible fitness costs for any genotype, equation 3 in the deterministic model predicts that without selection pressure from phage (*V(t) = 0*), the frequency of CRISPR+ cells should remain constant. However, in the non–-phage-treated conditions there was variation from the zero baseline in the CRISPR+ population frequency (Supplementary Fig. S2c). Specifically, we observed a decrease in the frequency of CRISPR immune bacteria when ${f}_{\Delta }$ was positive and C^+^ ≤ *R*^+^ (one-tailed *t*-test with FDR correction*, P* < .005) and an increase in their frequency when ${f}_{\Delta }$ was negative and *C*^+^ ≥ *R*^+^*(P* < .005).

This deviation from zero in the non-phage treated conditions suggests that our simple model does not describe the full picture of the background selective pressures acting on the different genotypes during co-culturing. For example, we ignored fitness costs for CRISPR-Cas immunity and surface modification–based resistance [9, 10] in order to be able to analyse the model analytically. The relative costs of CRISPR immunity and surface modification resistance has been shown to vary with environment, for example coexistence with other pathogens amplifies the fitness trade-off of receptor mutation [32]. As previously stated, simulations to explore the effect of differing costs, associated with immunity or resistance, on the ability of ${f}_{\Delta }$ to predict changes in the CRISPR+ population showed that provided the costs were not too large, the predictive power of ${f}_{\Delta }$ holds (Supplementary Fig. S1). To make the experimental results more robust we decided to control for these effects, and thus calculated the change in CRISPR+ population frequency from the baseline of the non-phage treated samples (Fig. 2c), thereby making our experimental results more comparable to the mathematical assumptions in the model presented previously. This frequency change is used to assess $\frac{d{N}^{+}}{dt}$ from the model equations (3). As predicted by the model, when ${f}_{\Delta }$ is positive the CRISPR+ population is selected for and increased in frequency, and vice versa when ${f}_{\Delta }$ is negative. This relationship appears to be modulated by the relative frequencies of immune or surface-based resistant cells within the CRISPR+ population, with the greatest change in the CRISPR+ population frequency being seen when the immune cells outnumber the resistant CRISPR+ cells.

To explore the empirical evidence quantitatively a general linear statistical model was created and showed that both the initial value of ${f}_{\Delta }$ and the initial ratio of immune to resistant CRISPR+ cells are associated with the change in frequency of the CRISPR+ population (adjusted *R*-squared = 0.87, *F*_3, 30_ = 76.52*, P* = 3.709e-14). Increasing ${f}_{\Delta }$ increases the change in the frequency of CRISPR+ cells (mean difference ± SE = 1.571126 ± 0.173532, bootstrapped confidence intervals = 1.19242–2.561325*, P* = 4.39e-10), and to a lesser extent, increasing the ratio of immune cells compared to resistant cells in the CRISPR+ population also increases the selection for the overall CRISPR+ population (mean difference ± SE = 0.044975 ± 0.008683, bootstrapped confidence intervals = 0.0270075–0.0564525*, P* = 1.40e-05). The effect of the initial frequency of CRISPR+ cells in the population did not show a significant effect on the selection for the CRISPR+ cells over the course of the experiment. This supports the mathematical model [equation (3)] that whether CRISPR+ cells increase or decrease in frequency in the short term does not depend on their initial frequency.

**Figure 3:**
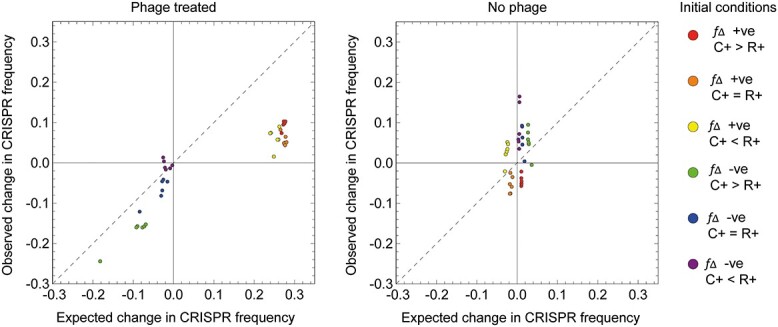
The observed change in CRISPR frequency is well predicted by our theoretical model in the presence of phages. Initial density of bacteria$N(0)={10}^6,$ and initial frequencies of the different cells given by the observed frequencies at $t=0$. On the left we show the output of the model (expected versus observed) in the presence of the phage ($V(0)={10}^4$ in the model) and on the right without the phage ($V(0)=0$). Parameter values: $r=0.5,{m}_{\,b}={m}_{\,v}=0,K={10}^9,\alpha ={10}^{-9},\mu =0,B=100,{c}_R=0.05,{c}_I=0,A=0$.

By including the measured initial strain genotype frequencies in the model, the observed change in CRISPR+ cell frequency holds the qualitative prediction that the sign of ${f}_{\Delta }$ determines whether the CRISPR+ population is selected for, and increases in frequency, or is selected against (Fig. 3). This observation demonstrates that over the short term this deterministic mathematical model can provide a qualitative prediction for the outcome of the biological experiments.

## Discussion

Modelling how subpopulations with CRISPR-Cas systems are selected for in mixed populations (namely isogenic populations with and without CRISPR-Cas defence systems) is an important piece of the puzzle for understanding how the possession of a CRISPR-Cas system, as well as other phage defence systems, may be fluid in a population under the “pan immune model” [22]. Phylogenetic and experimental evidence supports both the frequent gain of CRISPR-Cas systems by horizontal gene transfer [6, 23–25, 39] and the relative ease at which these systems are lost when costly [12, 13, 17, 18, 20, 21]. In this study we sought to address the current gap in evidence of how, once a CRISPR-Cas system has been gained by a subpopulation, it may be selected for in a population lacking CRISPR-Cas.

Previous experimental work in *P. aeruginosa* has shown that mutation of phage receptors is frequently utilised in preference to CRISPR-Cas immunity [10, 29–31]. Computational modelling has similarly suggested that the range of conditions under which CRISPR-Cas becomes a dominant defence mechanism are limited [26, 33, 34]. Mathematically modelling how CRISPR+ cells spread within a population of CRISPR− sensitive bacteria in the presence of phage suggests that there will be a frequency dependence on the success of the population growth—at lower initial frequencies the CRISPR+ bacteria are less likely to become established [26]. However, for CRISPR-Cas to be frequently successfully horizontally transferred, as suggested by the pan-immune model, subpopulations with this new defence system must be robustly selected for in the total population from an initially extremely rare phenotype. Experimental evidence to address this part of the theory is currently lacking. Evidence from *Vibrio* species shows that this evolutionary turnover of phage defence systems can be rapid [40], meaning that such loss, gain, and population establishment dynamics will play out many times within wild populations of bacteria.

To address these questions, we first created a mathematical model to consider the drivers of selection in a mixed population of a bacterial species with and without CRISPR. This model considers 5 genotypes that make up such a population: CRISPR+ cells that are either sensitive to the phage, resistant through the mutation of surface receptors, or immune via the acquisition of phage-targeting spacers; or CRISPR− cells that are either sensitive to or have surface-receptor–based resistance to the phage. By using a population of mixed genotypes, we were able to study the selection pressures that may select for or against a subpopulation in possession of a CRISPR-Cas system without adding the complexity of time to emergence of resistance or immune phenotypes. The output of this model suggests that difference in prevalence of resistance or immunity in CRISPR+ vs in CRISPR− cells (described as the term ${f}_{\Delta }$) is the main driver of whether the CRISPR+ subpopulation is selected for or against. When ${f}_{\Delta }$ is positive, CRISPR+ cells will increase their frequency in the population and when ${f}_{\Delta }$ is negative, CRISPR+ subpopulation will decrease in frequency.

To test this model empirically we designed a system in which 5 *P. aeruginosa* PA14 strains, each representing 1 of the 5 genotypes in the deterministic model, were edited to include a unique DNA sequence in their genome—genetically tagging them, meaning the relative proportion of each strain in the overall population could be quantified by qPCR amplification. This quantification technique has previously been used to follow selection in mixed species populations without the addition of unique sequences [32] and follows a larger trend of barcoding and sequencing techniques as a useful tool for tracking populations during evolution experiments (reviewed in [41]). The selection experiment was restricted to 24 hours of co-culturing, reducing the potential for the emergence of newly evolved phenotypes during the experiment, as barcoding and qPCR quantification allows only the measurement of selection for/against each pre-assigned genotype as a stand-in for phage resistance/sensitivity phenotypes, not the measurement phenotype frequencies directly. However, given the short time course of the experiment, we would expect the levels of mutation leading to changes in resistance/immunity/sensitivity phenotypes to be negligible.

These empirical results support the deterministic model showing that when the subpopulations of strains were combined in defined ratios to give a positive ${f}_{\Delta }$ value then the overall CRISPR+ population was selected for over the experiment and vice versa for when ${f}_{\Delta }$ was negative. Additionally, the results show that this relationship is modified by the ratio of phage-immune to phage-resistant cells within the CRISPR+ subpopulation, with the magnitude of selection being increased when immune cells outnumber resistant cells. By contrast, the initial frequency of CRISPR+ cells alone did not correlate well with the selection for or against CRISPR+ cells.

Our results suggest that the factor driving selection for CRISPR+ is whichever subpopulation has a higher frequency of cells that are protected against phage lysis: that is, if the frequency of CRISPR+ cells that are resistant or immune is higher than the frequency of CRISPR−- cells that are resistant, then ${f}_{\Delta }$ will be positive and the CRISPR+ subpopulation will be selected for. Therefore, when a population that has newly acquired a CRISPR-Cas system via HGT is becoming established, if the CRISPR+ population is able to gain higher frequencies of phage resistance/immunity (${f}_{\Delta }$ positive) then it will be able to compete with and establish in an isogenic population lacking CRISPR-Cas. When considering that the CRISPR+ population has two evolutionary pathways to take to gain protection against phage (resistance or immunity) compared to only resistance in the CRISPR− population, this may be a selection process that is regularly repeated in a population frequently losing and gaining defence systems. Other scenarios of HGT events could also be possible in populations frequently losing and gaining CRISPR-Cas beyond what was explored in the study reported in this paper. For example, CRISPR− cells could gain a CRISPR-Cas system already replete with a phage-targeting spacer, creating a population of either immune or both resistant and immune cells. Thus, our evidence reported here provides support to the pan-immune model that defence system possession may be fluid in populations of prokaryotes. This study furthers our understanding of how CRISPR-Cas systems may have successfully spread and been selected for during their evolutionary history. Indeed, in our model if the costs of CRISPR are negligible, then CRISPR itself is not a target of selection, that is, whether a cell carries a CRISPR system or not does not impact its fitness. Instead, what is the target of selection is whether cells have some form of resistance (either genetic or CRISPR-immunity) to the phage. During a phage epidemic, the population will become enriched for cells that have some form of resistance. Thus, if a greater frequency of CRISPR cells carry some form of resistance than non-CRISPR cells, CRISPR will increase in frequency as a by-product of selection for phage resistance. That is, CRISPR cells will increase in frequency due to indirect selection: hitchhiking upon selection for general resistance to phage.

The pan-immune model, in which defence systems are frequently lost and horizontally transferred, is relevant to the evolutionary trajectories of general defence systems, not just CRISPR-Cas. Furthering our understanding of how CRISPR-Cas HGT events can be successful, and become established within populations, may generalise to the HT of other phage defence systems. It is likely that all defence systems will not be equally permissible to such flexible acquisition and loss across a population. For example, upon transfer of restriction modification defence systems, the unmethylated genomic DNA of the new host may be targeted, creating autoimmunity [42, 43]. Potential incompatibilities are less likely for CRISPR-Cas systems, unless a self-targeting spacer is present in the CRISPR array. Somewhat paradoxically, the possession of phage defence systems such as CRISPR-Cas can act as a barrier to horizontal gene transfer [44]. This would not pose a problem to a host lacking such defence systems or with previously degraded defence genes but could prevent the continual acquisition of defence systems when horizontal gene transfer is common in the population.

With the continuous discovery of new bacterial defences has also come the recognition that defence systems frequently cluster in mobile “defence islands” [45]. These islands act as runways, providing an easy landing and takeoff point from the genome, facilitating HGT of defence systems. The role of HGT in the evolutionary dynamics of defence systems it seems, is inarguable. But, as our knowledge of the number, prevalence, and mobility of defence systems increases, it reveals gaps in our current understanding of the underlying evolutionary consequences and the interplay with population dynamics that determine the spread and maintenance of defence systems [46]. How do acquired defence systems integrate into the host, especially with pre-existing defence systems? How are horizontally acquired systems regulated to reduce cost of expression? Under what selective conditions would we expect defence systems to persist and at what frequency do we expect them to be lost? Answering questions such as these will help us to understand the natural prevalence and diversity of defence systems, the integration of defence systems with the host and with each other, and the implications of acquired defence systems in bacteria–-phage coevolutionary dynamics.

## Supplementary Material

Third_Elliott_et_al_Supplementary_clean_wrae108

## Data Availability

All data generated or analysed during this study are included in this published article as a Supplementary Data file: https://doi.org/10.5281/zenodo.10558270.
