# Supplementary material for: Conditions for the spread of CRISPR-Cas immune systems into bacterial populations
Source: ISME J. 2024 Jun 19;18(1):wrae108. doi: 10.1093/ismejo/wrae108 (PMC11285788; doi:10.1093/ismejo/wrae108)
Supplement: Third_Elliott_et_al_Supplementary_clean_wrae108 [file third_elliott_et_al_supplementary_clean_wrae108.docx]

**Supplementary Information**

**Conditions for the spread of CRISPR-Cas immune systems into bacterial populations.**

Authors: Josie F. K. Elliott^1,2#^, David V. McLeod^3,4#^, Tiffany Taylor^1^, Edze R. Westra^2^, Sylvain Gandon^5^, Bridget N. J. Watson^2#^

^1^ Milner Centre for Evolution, Department of Life Sciences, University of Bath, Claverton Down, Bath BA2 7AY, United Kingdom

^2^ ESI, Biosciences, University of Exeter, Cornwall Campus, Penryn TR10 9FE, UK

^3^ Département de mathématiques et statistique, Université de Montréal, Montréal, Canada

^4^ Institute of Ecology and Evolution, Universität Bern, Bern, Switzerland

^5^ CEFE, CNRS, Univ Montpellier, EPHE, IRD, Montpellier, France

# to whom correspondence should be addressed

**
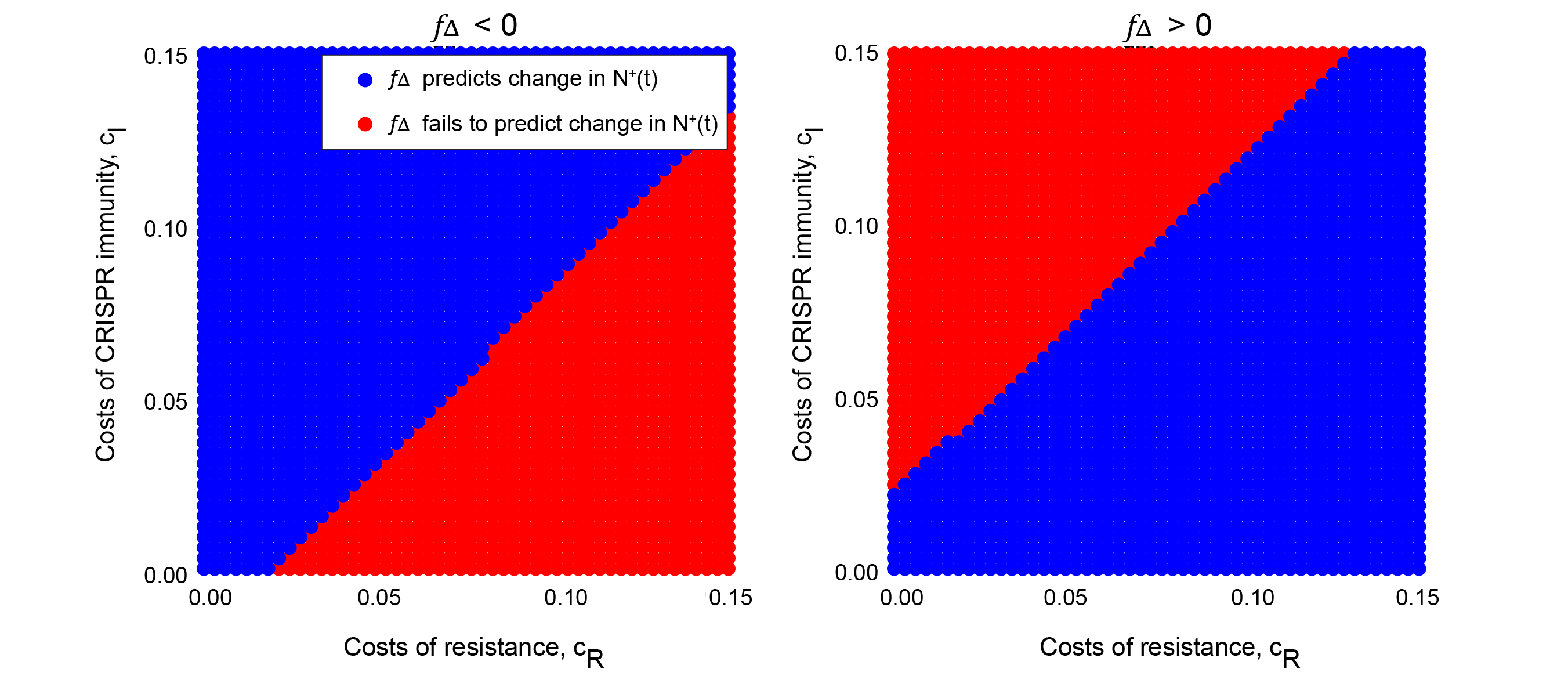
**

*Supplementary figure 1*: **Varying costs of immunity and resistance on the predictive power of** $\boldsymbol{f}_{\boldsymbol{\Delta}}\boldsymbol{.}$Only when costs of resistance and/or immunity are high and $f_{\Delta}$ is small in magnitude, will the sign of $f_{\Delta}$ at time t = 0 fail to predict the change in CRISPR frequency, $N^{+}(t$). In both panels, blue (resp. red) indicates that the sign of $f_{\Delta}$ at t = 0 predicts (resp. fails to predict) whether CRISPR increases ($f_{\Delta}$ > 0) or decreases ($f_{\Delta}$ < 0) in frequency from t = 0 to t = t_end_, where t_end_ is chosen to be the time at which the phage epidemic ends (i.e., the density of phage has dropped below it’s initial amount). We end the simulations at t = t_end_ because in the absence of phage, costly genetic resistance and CRISPR immunity will be selected against and lost from the population. Both panels used parameter value $r=0.5, \mathcal{m}_{b}=\mathcal{m}_{v}=0, K={10}^{9}, \alpha={10}^{-9},\mu=0, B=100, A=0$, with initial conditions N(0) = 10^6^, V(0) = 10^4^, with the frequency of CRISPR being 1/2, while the frequency of phage-protected CRISPR cells with genetic resistance is 1/2. In the left panel, $f_{\Delta}$ = -0.01, while in the right panel, $f_{\Delta}$ = 0.01. Increasing the magnitude of $f_{\Delta}$ at time t = 0 will increase the parameter space for which $f_{\Delta}$ predicts the change in $N^{+}(t$).


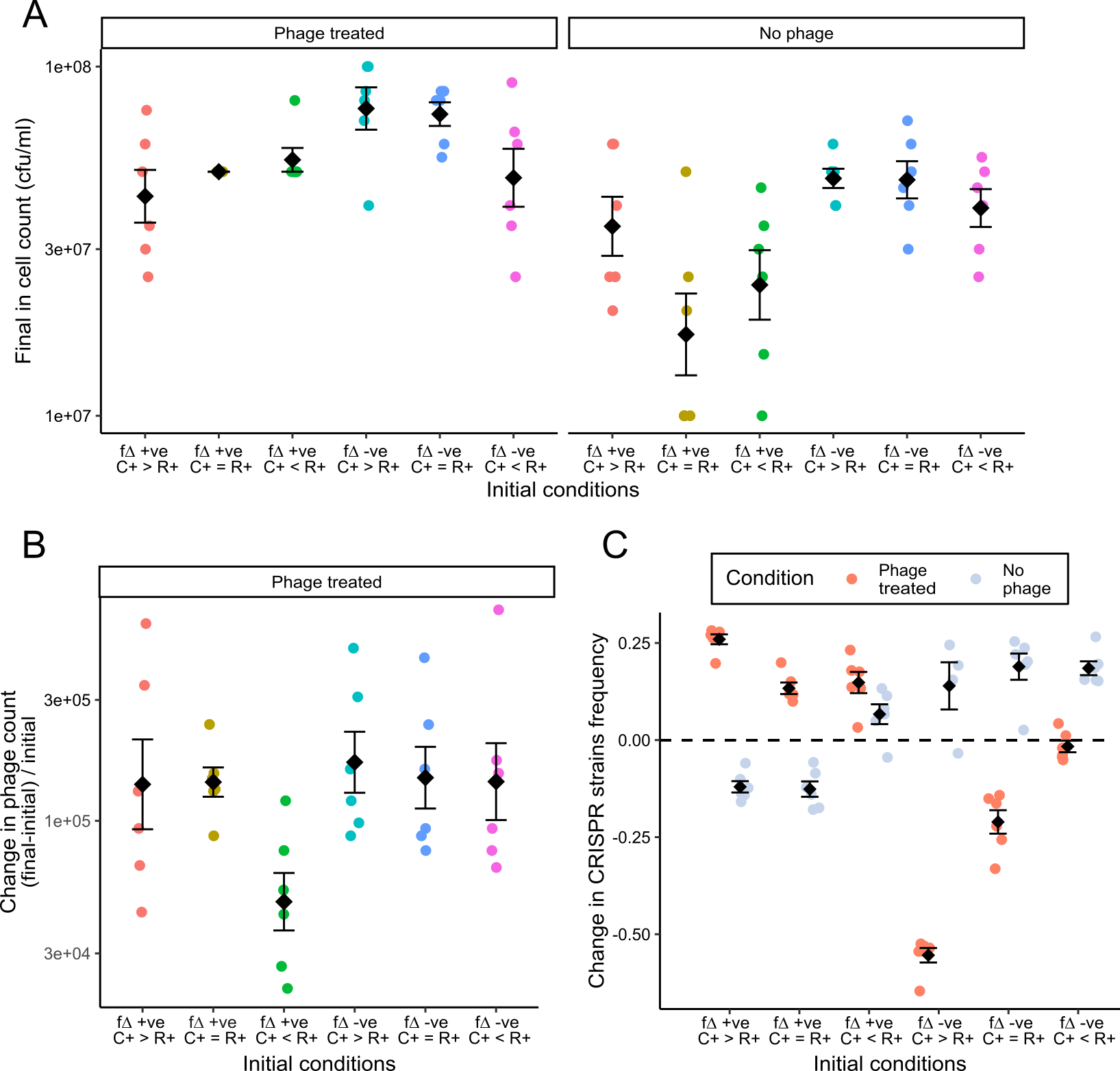


Supplementary figure 2. (a) Final cell counts for each experiment are shown, coloured according to the initial treatment conditions with circle showing individual repeats (N=6) with black diamond shapes denoting the mean and error bars showing the interquartile range. (b) The change in phage titre (pfu/ml) over the 24-hour selection experiment (final phage titre – initial phage titre / initial phage titre). Formatting same as (a). (c) Relative change in CRISPR+ population frequency (final CRISPR+ frequency – initial frequency / initial frequency). Data is coloured by phage selection conditions with circles representing repeats (N=6) and black diamonds showing mean with interquartile error bars. In the non-phage treated conditions CRISPR+ population frequency there was variation from the zero baseline. In the $f_{\Delta}$ positive C^+^ < R^+^, C^+^ = R^+^ experiments the mean was significantly different from the zero baseline (one-tailed t-test with FDR correction, p = 0.001, 0.003 respectively with the $f_{\Delta}$ positive C^+^ < R^+^ condition at p = 0.057). Similarly, in the $f_{\Delta}$ negative C^+^ > R^+^, C^+^ = R^+^ experiments the mean was significantly different from the zero baseline (p = 0.001, 0.004 respectively with the $f_{\Delta}$ negative C^+^ > R^+^ condition at p = 0.105). This informed the choice to adjust the change in CRISPR strains frequency to be normalised against the no phage conditions (Fig. 3).

**Supplementary Information: Interactions between horizontal gene transfer and** $\boldsymbol{f}_{\boldsymbol{\Delta}}$

Here we consider the effect of horizontal gene transfer (HGT) of CRISPR-Cas systems on the change in $f_{\Delta}$. We suppose that for each instance of HGT, a ‘donor’ cell that is CRISPR positive copies CRISPR and transfers the copy to a ‘recipient’ cell that is CRISPR negative. There are three possible types of HGT, which we detail below. For clarity we assume that each HGT “event” will reduce the population density of the recipient cell by a small amount $\delta$, and cause a concomitant increase in population density of the relevant class of CRISPR positive cell.

1. A sensitive cell (i.e., $S^{-}$), acquires CRISPR from a cell that does not carry the spacer encoding for resistance to the focal phage (i.e., $S^{+}$, $R^{+}$). This will change the population densities such that:

$$\left( S^{-},S^{+} \right)\to\left( S^{-}- \delta, S^{+}+ \delta\right).$$

Consequently, the change in $f_{\Delta}$ due to HGT is:

$\Delta f_{\Delta}= -\frac{\delta}{NN^{+}(1-N^{+})}\left( \frac{R^{+}+C^{+}}{R^{+}+ C^{+}+ S^{+}}\left( 1 - N^{+} \right)+\frac{R^{-}}{R^{-}+ S^{-}} N^{+} \right)\mathcal{+ O}\left( \delta^{2} \right)$,

where $N^{+}$ is the frequency of CRISPR and $N$ is the total density of cells. Since

$\Delta f_{\Delta}< 0$, in this case HGT decreases $f_{\Delta}$.

1. A cell with genetic resistance acquires CRISPR,

$$\left( R^{-},R^{+} \right)\to\left( R^{-}- \delta, R^{+}+ \delta\right).$$

In this case, the change in $f_{\Delta}$due to HGT is:

$$\Delta f_{\Delta}= \frac{\delta}{NN^{+}(1-N^{+})}\left( 1-\frac{R^{+}+C^{+}}{R^{+}+ C^{+}+ S^{+}}\left( 1 - N^{+} \right)-\frac{R^{-}}{R^{-}+ S^{-}} N^{+} \right)\mathcal{+ O}\left( \delta^{2} \right).$$

Since

$$\frac{R^{+}+C^{+}}{R^{+}+ C^{+}+ S^{+}}\left( 1 - N^{+} \right)+\frac{R^{-}}{R^{-}+ S^{-}} N^{+}\leq1-N^{+}+N^{+}=1,$$

it follows that $f_{\Delta}>0$, and so in this case HGT increases $f_{\Delta}.$Indeed, an identical transition happens if resistant cells without CRISPR ($R^{-}$) acquire CRISPR with a spacer (and so carries both genetic resistance and immunity), as this will decrease the density of resistant cells that do not carry CRISPR by $\delta$ (i.e., $R^{-}\to R^{-}- \delta$) and increase the density of CRISPR positive cells with some form of resistance by an amount $\delta$ (i.e., $C^{+}+ R^{+} \to C^{+}+ R^{+} + \delta$). Although we do not explicitly include an equation for cells that carry both genetic resistance and CRISPR immunity in the model of equations  (1), if resistance and immunity provide similar protection and are of negligible cost, the model of equations (1)  captures the dynamics of a model with an additional class of cells that carry both CRISPR immunity and resistance, as these cells will follow a similar dynamical equation to $C^{+}$.

1. A sensitive cell acquires CRISPR with a spacer,

$$\left( S^{-},C^{+} \right)\to\left( S^{-}- \delta, C^{+}+ \delta\right).$$

In this case, the change in $f_{\Delta}$ due to HGT is:

$$\Delta f_{\Delta}= \frac{\delta}{NN^{+}(1-N^{+})}\left( 1 - N^{+}-\frac{R^{+}+C^{+}}{R^{+}+ C^{+}+ S^{+}}\left( 1 - N^{+} \right)-\frac{R^{-}}{R^{-}+ S^{-}} N^{+} \right)\mathcal{+ O}\left( \delta^{2} \right).$$

This can be either positive or negative. Notice that we can write

$\Delta f_{\Delta}=\frac{\delta}{N N^{+}(1-N^{+})}\left( 1 - N^{+}-N_{R}-f_{\Delta}(1-2N^{+}) \right)$

where $N_{R}=\frac{R^{-}+ R^{+}+ C^{+}}{N}$ is the total frequency of resistance (genetic or CRISPR) in the population. Thus when $\Delta f_{\Delta}\approx0$, there are two cases:

1. If the frequency of resistance and CRISPR are high, $N^{+}+ N_{R}> 1$, HGT will produce negative $f_{\Delta}$,
2. if the frequency of resistance and CRISPR are low, $N^{+}+ N_{R}< 1$, HGT will produce positive $f_{\Delta}.$

The three cases above examine the situation where *given* HGT of the specified type occurs, what is the effect on $f_{\Delta}$. However, clearly the probability of which type of HGT occurs will depend upon the composition of the population, as generally we would expect the likelihood to be proportional to the population densities. In particular, the rate at which the different cases occur will be proportional to:

Case 1: $S^{-}\left( S^{+}+R^{+} \right)$, since $S^{-}$ is the recipient and $S^{+}$ or $R^{+}$ are the donors.

Case 2: $R^{-}\left( S^{+}+R^{+} \right)$, since $R^{-}$ is the recipient and $S^{+}$ or $R^{+}$ are the donors. Or $R^{-}C^{+}$if $R^{-}$ is the recipient and $C^{+}$ is the donor.

Case 3: $S^{-}C^{+}$, since $S^{-}$ is the recipient and $C^{+}$is the donor (or $R^{-}$).

Thus, for example, if resistance (either genetic or spacer) is rare, as might be expected in the early part of a phage epidemic, then HGT will be more likely to induce $S^{-}\to S^{+}$ transitions (case 1), and so HGT will have a net negative effect on $f_{\Delta}.$If instead resistance (either genetic or spacer) is more abundant, then $S^{-}\to C^{+}$or $R^{-}\to R^{+}$ transitions may also be common, depending upon whether resistance is more commonly linked to CRISPR, or if it is genetic, and in this case, HGT can have a net positive or negative impact on $f_{\Delta}$.

To put this all together, assume the rate constants controlling HGT are independent of donor and recipient. Then the total change in $f_{\Delta}$ due to HGT owing to each of these cases is proportional to

$$S^{-}\left( S^{+}+ R^{+} \right)\times\left( \text{Case 1} \right)+ \left( R^{-}C^{+}+ R^{-}\left( S^{+}+ R^{+} \right) \right)\times\left( \text{Case 2} \right)+S^{-}C^{+}\times\left( \text{Case 3} \right)=\frac{R^{-}S^{+}- R^{+}S^{-}}{NN^{+}}$$

And so if

$$\frac{R^{-}}{S^{-}}>\frac{R^{+}}{S^{+}}$$

HGT increases $f_{\Delta}$ otherwise HGT decreases $f_{\Delta}$.
